# Butyrate Induced Cell Cycle Arrest in Bovine Cells through Targeting Gene Expression Relevant to DNA Replication Apparatus^1^

**DOI:** 10.4137/grsb.s465

**Published:** 2008-03-17

**Authors:** Cong-jun Li, Robert W. Li

**Affiliations:** Bovine Functional Genomics Laboratory, Animal and Natural Resources Institute, ARS, USDA

**Keywords:** butyrate, cell cycle, DNA replication, gene expression, ORC

## Abstract

Using real-time RT-PCR and Western blot analysis in bovine kidney epithelial cells, we systematically investigated the effects of butyrate on patterns of gene expression relevant to DNA replication apparatus. The real-time PCR and Western blot data generally confirmed previously reported microarray data. Of the five genes tested by quantitative RT-PCR, CDKN1A (p21^waf1^) was up regulated, CDC2/cdk1, MCM6, ORC1L were down regulated, while ORC3L expression remained unchanged following butyrate treatment. Also consistent with RT-PCR results, Western blot analysis confirmed that butyrate up-regulated cyclin-kinase inhibitor p21^waf1^ in a does-dependent manner. In contrast, butyrate treatment had no effect on the expression of ERK 1/2 proteins. Also consistent with mRNA results, ORC1 and MCM3 proteins were down-regulated by butyrate treatment, while ORC2 protein remained unchanged. The present results suggest that ORC1, not ORC2 or ORC3, along with MCM proteins play a critical role in regulating the initiation of DNA replication and cell cycle progression in MDBK cells and are targets of butyrate regulation.

## Introduction

In cattle, few definitive studies, if any at all, have sought to address the capabilities of nutrients to modulate gene expression and proteomic outcomes as a means of arresting metabolic stress. Short-chain fatty acids (SCFAs, i.e. acetate, propionate, and butyrate) are formed during microbial fermentation of dietary fiber in the gastrointestinal tract of mammalian species and then are directly absorbed at the site of production. SCFAs contribute up to 70% of the energy requirements of ruminants (Bergman, 1990). Although acetate and propionate hold a prominent position in providing energy to ruminant metabolism, butyrate, which is present in relative low concentrations, seems to be involved in metabolism beyond its role as a nutrient. Ruminant species metabolize SCFAs to fulfill up to 70% of their nutrient energy requirements. Beyond their nutritional impact; SCFAs, especially butyrate, modulate cell differentiation, proliferation, motility, and in particular, induce cell cycle arrest and apoptosis. However, a majority of previous studies were focused on the cancer therapeutic potential of butyrate and thus used cancer cell lines as research models (Myzak and Dashwood, 2006). The cell cycle regulatory effects of butyrate at the cellular and molecular levels in normal bovine cells have not been studied thus far, but would serve as a principle launching point to validate the need for further study of this phenomena in cattle. Because short-chain fatty acids are common nutrients, understanding their important biological functions additional to a simple energy supply will certainly help us to understand critical control points in the cell cycle that could lead to improvements in the efficient production of food animals. For example, during periods of naturally encountered stresses, such as the weaning period when production and uptake of short-chain fatty acids are significantly increased, short-chain fatty acids-induced apoptosis and cell cycle arrest may affect growth slumps of young cattle. Minimizing the duration of growth slumps has the potential to affect the industry significantly. In addition, MDBK as an established bovine cell line with inducible apoptosis and cell cycle regulatory events certainly is ideal and an invaluable tool for functional genomic studies on homeostasis in animals.

At physiologic concentration, butyrate regulates the expression of individual genes using at least three different mechanisms: (1) through induction of cis- and trans-acting butyrate-dependent transcription factor for selected genes, (2) by inhibition of histone deacetylation and attendant chromatin remodeling and (3) by affecting turnover of mRNAs (Parab et al. 2007; Myzak et al. 2006). The major biochemical change that occurs in cells treated with butyrate and other histone deacetylase (HDAC) inhibitors is the global hyperacetylation of histones (Riggs et al. 1977). In our previous studies, a normal bovine kidney epithelial cell line (Madin-Darby bovine kidney; MDBK) was used to investigate the cell cycle regulatory and apoptotic effects of butyrate. Butyrate not only induced apoptosis but also induced cell cycle arrest at the G1/S boundary in MDBK cells. We used sodium butyrate, an important nutrition component for cattle and a histone deacetylase inhibitor to show that butyrate treatment was able to stop MDBK cell proliferation and cells undergo G1/S arrest. Our data also indicated that DNA replication was blocked at a very early stage of S-phase by butyrate treatment (Li and Elsasser, 2005). Since butyrate blocks cell cycle progression at very early stage of S-phase, it is reasonable to assume that genes directly related to initiation and regulation of DNA replication and cell cycle progression may be targeted by butyrate. Using high-density microarrays to examine the global gene expression and global gene expression profiles of the bovine kidney epithelial cells, we discovered 450 genes that were significantly regulated by butyrate with a median False Discovery Rate (FDR) = 0%. The majority of these genes was repressed by butyrate and was associated with cell cycle control (Li and Li, 2006). The microarray data is available as accession GSE3970 in the Gene Expression Omnibus repository at the National Center for Biotechnology Information (http://www.ncbi.nlm.nih.gov/geo/).

Cell-cycle arrest at G1 phase induced by butyrate suggests that a common responding element in genes responsive to the treatment of butyrate is required for progression of phase G1. This observation is also consistent with the previous report that the inhibition of G1 progression by butyrate is not restricted to a specific mitogenic signaling pathway (Charollais et al. 1990), but may also include the inhibitory effect on initiation of DNA replication. Especially, the effects of butyrate on patterns of gene expression relevant to DNA replication apparatus are probably the causes of inhibition of initiation of DNA replication. To determine the epigenetic effects of physiologic concentrations of butyrate on cellular level of proteins, which are the putative targets of butyrate induced cell cycle arrest, our effort in this study was focused on the initiation of DNA replication apparatuses regulated by butyrate. We systematically investigated the expression regulation of the DNA replication regulatory genes by butyrate using both real-time RT-PCR and Western blot analysis in bovine kidney epithelial cells. Our results confirmed and extended our earlier findings of butyrate-regulated gene expression in our earlier publication (Li and Li, 2006). The present results suggest that ORC1, not ORC2 or ORC3, along with MCM proteins play a critical role in regulating the initiation of DNA replication and cell cycle progression in MDBK cells.

## Materials and Methods

### Cell culture and cell treatments

The Madin-Darby bovine kidney epithelial cells (MDBK, American Type Culture Collection, Manassas, VA., and Catalog No. CCL-22) were cultured in Eagle’s minimal essential medium supplemented with 5% fetal bovine serum (Invitrogen, Carlsbad, C.A) in 25 cm^2^ flask with medium renewal twice per week. Cell cultures were maintained in a water-jacked incubator with 5% CO_2_ at 37 °. Sub-cultivations were performed when cells attained 80 to 90% confluence, according to the product information supplied by American Type Culture Collection. Cells were used for treatment testing at approximately 50% confluence, during the exponential phase of growth.

Sodium butyrate (Calbiochem, San Diego, C.A) was prepared as 1 *M* stock by dissolving it in ultra pure de-ionized water (tissue culture grade, Advanced Biotechnologies Inc., Columbia, MD). Final concentrations of sodium butyrate treatment ranged from 2.5 to 10 m*M*. Adding up to 10 m*M* sodium butyrate into cell culture medium did not cause measurable pH changes. Each treatment was performed in duplicate. After 24 hours of sodium butyrate treatment, cells were collected by trypsinization. Viability of cells was determined by Trypan Blue stain exclusion (Invitrogen).

5-Bromo-2′-deoxyuridine (BrdU) incorporation labeling and Flow cytometric analysis of cells.

Cells were labeled with BrdU using BrdU Flow kits (BD Pharmingen, San Diego, C.A). To pulse label the cells, 10 μl of BrdU solution (1 mM BrdU in PBS) was carefully added directly to each ml of culture media and cultured for 40 min. To immunofluorescent stain cells, cells were collected with trypsinization, fixed, and permeabilized with BD Cytofix/Cytoperm buffer, supplied with the kits. Cells were then treated with DNase to expose incorporated BrdU (30 μg of DNase to each sample, incubated at 37 °C for 1 hour). After washing with 1 ml of 1X BD Perm/wash buffer, cells were resuspended with 50 μl of BD Perm/Wash buffer containing diluted fluorescent (Fluorescent isothiocyanate, FITC) anti-BrdU antibody and incubated for 20 min at room temperature. After washing with BD Perm/Wash buffer, cells were resuspended in 20 μl of 7-AAD solution (supplied with kits) to stain the cellular DNA content. The amount of DNA was measured by the amount of dye taken up by the cells and indirectly by the DNA content. Flow cytometric analyses were performed, using a flow cytometry (FC500, Beckman Coulter Inc., Palatine, IL) and collected data was analyzed using Cytomics RXP (Beckman Coulter Inc.). At least 10,000 cells per sample were analyzed.

### Real-time RT-PCR

Real-time RT-PCR analysis was carried out with the IQ SYBR Green Supermix kit (Biorad) using 200 nM of each amplification primer Table 1 and the 1st-strand cDNA (100 ng of the input total RNA equivalents) in a 25 μl reaction volume. The amplification was carried out on an iCycler iQ^™^ Real Time PCR Detection System (BioRad) with the following profile: 95 °C–60s; 40 cycles of 94 °C–15s, 60 °C –30s, and 72 °C–30s. The melting curve analysis was performed for each primer pair. Expression levels of β-actin remained constant (within 0.5 Ct between samples) and were used as endogenous controls. Relative gene expression data was calculated using the 2^−ΔΔ^*^C^*^T^ method (Livak and Schmittgen, 2001).

### Preparation of cell extracts and western blot analyses

Cells were collected from culture flasks by trypsinization. Cells were then washed with complete medium including a 5% fetal bovine serum, and followed by PBS twice. Cells were extracted with 10 volumes of M-PER (Mammalian Protein Extraction Reagent, Pierce Biotechnology, Rockford, IL) supplemented with 150 m*M* NaCl and protease inhibitor cocktail (Protease inhibitor cocktail tablets, Boehringer Mannheim, GmbH, Germany) and then incubated on ice for 10 min before centrifugation (1500 g, 5 min at 4 ° in Eppendorf microfuge) to eliminate debris. SDS-PAGE was done in a 4 to 20% gradient polyacrylamide gel (Invitrogen) under the reducing conditions suggested by the manufacturer. Pre-stained molecular weight standards were included (SeeBlue-plus2, Invitrogen). Separated proteins were transferred to pure nitrocellulose membrane (Protran, 0.2 μm, Schleicher and Schuell, Dassel, Germany). Western blot analyses were performed using anti- cdc6 antibody, anti-acetyl histone3 (H3), and anti p21 antibody (Cell Signaling Technology, Beverly, MA). Secondary antibodies used were horseradish peroxidase-conjugated anti-mouse or anti-rabbit IgG antibodies (Pierce Biotechnology, Rockford, IL). Membranes were blocked first with 5% of fat-free dry milk in PBS for one hour and followed by incubation with antibodies specified (1:2000 diluted in PBS plus 0.1% Triton 100). Membranes were then washed five times with PBS containing 0.1% Triton 100. Secondary antibodies (1:25000 diluted with PBS plus 0.1% Triton 100) were added and incubated for one hour. Membranes were washed 5 times with PBS containing 0.1% Triton 100. Immunoblots were exposed to SuperSignal West Pico Stable Peroxide solution with a luminol/enhancer (Pierce Biotechnology) according to the manufacturer’s instruction. Western blots were then scanned and analyzed using UN-SCAN_IT gel (V 61, Silk Scientific) to quantify the density of the bands.

## Results

### Butyrate induces cell cycle arrest and hyperacetylation of histone 3 in MDBK cells

We previously reported butyrate induced cell cycle arrest in MDBK cells (Li and Elsasser, 2005). Prior to mRNA and protein analysis, the butyrate induced cell cycle arrest was reconfirmed. As shown in the Figure 1A, after butyrate treatment for 24 h, cell population profiles changed significantly. Cells in S-phase were almost completely eliminated in 10 mM butyrate treated cell populations. This result confirmed our prior observation that cells were arrested at the G1/S boundary and DNA replication was blocked by butyrate treatment. We also confirmed the accumulation of acetylated histone 3 (H3) due to butyrate treatment (Fig. 1B). Monoclonal antibody against acetyl H3 was used to evaluate the histone deacetylase inhibitory activity of butyrate. As shown in Figure 1B, butyrate treatment induced accumulation of acetyl H3 in a does-dependent manner.

### Validation of microarray data by quantitative RT-PCR

Previous microarray data (Li and Li, 2006), indicated that butyrate induced significant changes in mRNA expression of genes directly involved in DNA replication (Table 2). DNA microarray analysis measures expressional changes in thousands of transcripts, and therefore, the statistical evaluation of this data is prone to type I errors (false positive results). We identified genes significantly regulated by sodium butyrate at a very stringent false discovery rate (FDR) = 0%. However, we think that validation of the initial microarray data by quantitative RT (reverse transcriptase)-PCR is necessary, wherever possible. The real-time PCR data generally confirmed the microarray analysis. Linear regression analysis demonstrated a strong positive correlation between the two technological platforms. Of five genes that we tested, CDKN1A (p21) was up regulated by butyrate, CDC2/cdk1, MCM6, ORC1L were down regulated, while ORC3L remained unchanged upon butyrate treatment.

### Protein expression analysis

**Figure 2:**
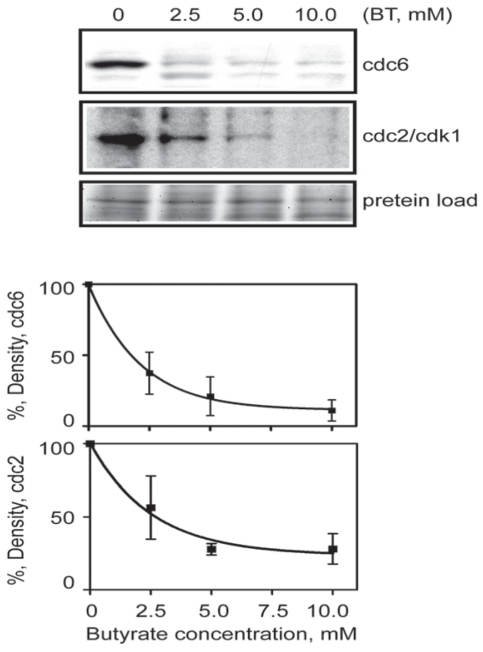
Butyrate treatment induced down regulation of cell cycle regulatory proteins cdc6 and cdc2/cdk1. Butyrate treated cells were extracted and proteins were separated on SDS PAGE. Western Blot analyses were performed with antibody against cdc6 and cdc2/cdk1. Western Blots from three experiments were quantified with UN-SCAN-IT software (Silk Scientific, Orem, Utah. U.S.A). The relative densities were measured and corrected with the protein density. Density of control band is presented as 100% (n = 3 per treatment). Data was statistically analyzed by ANOVA using GraphPad Prism 4.00 for Windows (GraphPad Software, San Diego, C.A., U.S.A). Data is presented as means +/− SEM.

**Figure 3:**
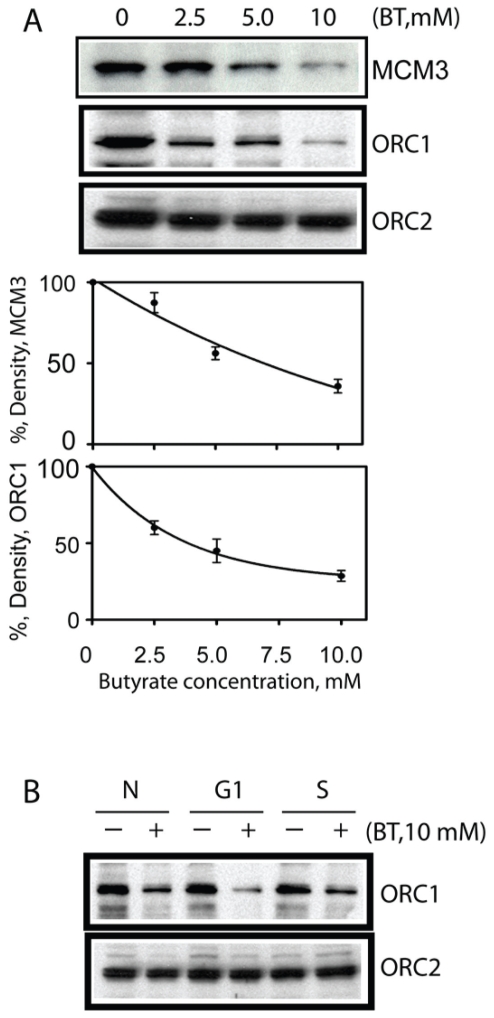
Butyrate treatment induced down regulation of MCM3 and ORC1 but has no effect on ORC2. Butyrate treated cells were extracted and proteins were separated on SDS PAGE. Western Blot analyses were performed with antibody against MCM3, ORC1 and ORC2. Western Blots from three experiments were quantified with UN-SCAN-IT software (Silk Scientific, Orem, Utah. U.S.A). The relative densities were measured and corrected with the protein density. Density of control band is presented as 100% (n = 3 per treatment). Data was statistically analyzed by ANOVA using GraphPad Prism 4.00 for Windows (GraphPad Software, San Diego, C.A., U.S.A). Data is presented as means +/− SEM. **A**) Normal cells were treated with or without butyrate. **B**) Cells were first synchronized by serum starvation for 72 hr (G1 phase) or released for 8 hrs after starvation (S phase) and then treated with or without butyrate. N: normal growing cells; G1: G1 phase cells; S: S phase cells.

**Figure 4:**
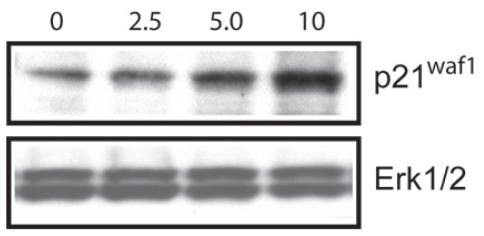
Butyrate treatment induced up-regulation of p21 but has no effect on ERK1/2. Butyrate treated cells were extracted and proteins were separated on SDS PAGE. Western Blot analyses were performed with antibody against p21 and ERK1/2. This figure is representative of two experiments. Western blot was quantified with UN-SCAN-IT software (Silk Scientific, Orem, Utah. U.S.A). The relative densities were measured and corrected with the protein density. Density of control band is presented as 100%. Data was statistically analyzed by ANOVA using GraphPad Prism 4.00 for Windows (GraphPad Software, San Diego, C.A., U.S.A).

A selection of proteins was examined using Western blot analysis based on availability of antibodies that recognize bovine proteins. These selected proteins included products of cell cycle regulatory genes such as p21, CDC2/CDK1, as well as proteins from DNA replication apparatus such as ORC1, ORC2 and MCM3. Overall there was a high correlation between mRNA and protein expression for all proteins analyzed. First, we examined cell cycle regulatory protein cdc6 and cdc2. As shown in Figures 2A and 3A, butyrate down-regulated CDC6 and CDC2/CDK1 in addition to ORC1 and MCM2 protein levels. Both of which were consistent with RT-PCR results. Interestingly, ORC2 protein expression did not change as a result of butyrate treatment. Since RT-PCR also confirmed that expression of ORC3 did not change upon butyrate treatment and only ORC1 mRNA was detected by microarray to be down-regulated by butyrate, it is reasonable to assume that among six subunits of ORC, ORC1 is the only gene was targeted by butyrate. We also synchronized cells using the serum starvation approach and monitored ORC1 protein expression. Under all three conditions (normal growing cells, cells synchronized at G1/G0 boundary and S phases), sodium butyrate inhibited ORC1 protein expression (Fig. 3B), suggesting the anti-proliferative and pro-apoptotic effects of butyrate were independent of cell cycle status. By comparison of Figure 2A and Figure 3A, decreasing levels of CDC6 and CDC2 are much deeper than ORC1 and MCM3. We suspect that the proteasome-dependent degradation of CDC6 and CDC2 may play some role as reported in an earlier study (Li and Elsasser, 2005). Finally, we tested and examined the protein level of p21^waf1^ and also examined the protein level of p21^waf1^, a negative gene expression regulatory gene. Butyrate up-regulated the cyclin-kinase inhibitor p21^waf1^ in a dose-dependent manner (Fig. 4). In contrary, butyrate treatment has no effect on expression levels of ERK 1/2 proteins.

## Discussion

The results we present here provide evidence for a direct link between the initiation of DNA replication and the cell growth regulatory pathways involving cell cycle progression. Initiation of eukaryotic DNA replication depends on the function of pre-replication complexes (pre-RC), one of its key components being the six subunits of the origin recognition complex (ORC). Eukaryotic DNA replication is a highly conserved process that begins with binding of a six-subunit ORC to DNA (Bell and Dutta, 2002). Proteins Cdc6 and Cdt1 (also called RLF-B) then load Mcm proteins 2 to 7 onto these ORC-chromatin sites to form pre-replication complexes (pre-RCs). MCM2 to MCM7 hexamers constitute the helicases that unwind the DNA. Pre-RCs are activated upon binding of Mcm10 protein (Wohlschlegel et al. 2002). CDC6 is then released by the cyclin-dependent protein kinase CDK2/cyclin A and replaced by CDC45 with the help of the protein kinases CDC7/Dbf4 and CDK2/cyclin E. DNA polymerase-DNA primase, which is escorted to the complex by Cdc45, then initiates RNA-primed DNA synthesis (S phase).

Recently, utilizing gene expression profiling, our studies indicated that butyrate induces many significant changes in the expression of genes associated with regulatory pathways that are critical to cell growth, immune response and signal transduction (Li and Li, 2006). Using the Ingenuity Pathway Knowledge base (Ingenuity® Systems, www.ingenuity.com), the functional category and pathway analysis of differential expressed genes in cells treated with butyrate were explored. The functional category and pathway analyses of the microarray data revealed that several canonical pathways (Cell cycle: G2/M DNA damage checkpoint; pyrimidine metabolism; Cell cycle: G1/S Checkpoint Regulation; and purine metabolism; insulin-like growth factor axis components) were significantly affected (Li et al. 2006). Interestingly, four pathways (Cell cycle: G2/M DNA damage checkpoint; pyrimidine metabolism; Cell cycle: G1/S Checkpoint Regulation; and purine metabolism) showed significant down-regulation. In addition, cell cycle checkpoint pathways (G2/M DNA damage checkpoint and G1/S Checkpoint regulation) are impaired due to the treatment of butyrate. Two pathways for critical regulation of purine and pyrimidine metabolism were also linked to butyrate induced biological effects.

For the first time, using microarray technique, MCM2, MCM3, MCM4, MCM5, MCM6 and ORC1, were found to be down-regulated by butyrate treatment (Table 3). In this study, we systematically investigated the expression regulation of selected DNA replication regulatory genes by butyrate using both real-time RT-PCR and Western blot analysis in bovine kidney epithelial cells. These selected genes included cell cycle regulatory genes such as p21, CDC2/CDK1, as well as genes for DNA replication apparatus such as ORC1, ORC2 and MCM3. Overall trends indicated there was a high correlation between mRNA expression and protein expression. The products of these genes all belong to pre-RC, which is a group of very important components of the DNA replication apparatuses. These proteins have been shown to be rate-limiting for the initiation of DNA replication in eukaryotic cell lines and are essential for the assembly of the pre-replication complex (Sun et al. 2002). Genes for MCM proteins 2, 3, 4, 5, and 6, as well as ORC1 (Origin Recognition Complex largest subunit) are significantly down-regulated by butyrate, suggesting that in some way butyrate treatment directly targets genes which are essential for the initiation of DNA replication.

The eukaryotic ORC selects the genomic sites where pre-replication complexes are assembled and DNA replication begins. In spite of a significant degree of conservation among ORC proteins from different eukaryotic sources, the regulation of their availability varies considerably in different model systems and cell types. Eukaryotic cells appear to regulate the assembly of functional ORC on chromatin sites through cell cycle-dependent modifications of one or more ORC subunits. ORC1 has been identified in previous studies as a primary control point in regulating the assembly of pre-replication complexes on mammalian chromosomes. First, ubiquitination of ORC1 was found to be an important regulation mechanism of initiation of DNA replication (Li and DePamphilis, 2002; Mendez et al. 2002). Second, the role for CDK1 (cyclin dependent protein kinase 1)/cyclin A in preventing the mammalian largest ORC subunit (Orc1) from binding to chromatin during mitosis was identified (Li et al. 2004). More intriguing, our results from this study indicate that there is another mechanism for the regulation of the function of ORC1 at the levels of RNA transcription and protein translation, which can be induced by butyrate treatment. Expression of bovine ORC1 gene is low after butyrate treatment, and the regulatory effects are dose dependent. In contrast, expression of bovine ORC2 and ORC3 genes do not appear to be similarly regulated. These results thus provide a direct link between the initiation of DNA replication and cell cycle arrest induced by butyrate. Moreover, ORC1 may be the most essential component of all replication complexes.

One universal feature of eukaryotic DNA replication is that the genome is replicated once and only once each time a cell divides. This is accomplished by restricting initiation events in at least two ways. First, pre-RCs that are assembled during the M to G1 phase transition are inactivated during S phase, and second, new pre-RCs cannot be assembled until G1 phase. In addition to blocking pre-RC assembly and activation at multiple steps such as the Cdc6, Cdt1, Mcm(2–7), and Cdk2 functions, the premier step in determining both where and when DNA replication begins is the assembly of functional ORC/chromatin sites, and this step appears to be regulated by inactivating ORC during the G1 to S phase transition (Li and DePamphilis, 2002; Tatsumi et al. 2003) and then preventing re-establishment of ORC activity until mitosis is completed and, in the metazoan, a nuclear membrane is reassembled (Li et al. 2004; Tatsumi et al. 2000; Thome et al. 2000). Although these studies suggest that ORC activity in mammalian cells is regulated by cell-cycle-dependent changes in Orc1 function (ORC cycle), it has been suspected for a long time that histone acetylation may also affects the initiation of DNA replication (Brown et al. 1991; Lipford and Bell, 2001; Simpson, 1990). However, at present neither the enzymes nor the steps involved are known (Iizuka et al. 2006). Even though it is likely that other gene products targeted by butyrate treatment may play functional roles in the cell cycle arrest, our results reported here indicated that epigenetic constraints imposed by chromatin structure, such as hyperacetylation induced by butyrate, may regulate aspects of DNA replication and origin selection by ORC proteins at the levels of RNA transcription and protein translation of ORC1.

Cyclin/cdk inhibitor p21^waf1^ is required for butyrate-mediated growth inhibition of human cancer cells. Butyrate and other SCFAs induce growth arrest in colon cancer cells through a process requiring the induction of the G1 cell cycle inhibitor p21^waf1^ (Archer et al. 1998). The activity of a variety of protein cyclins and their associated cyclin-dependent kinase (CDK) is required for the cell cycle progresses in mammalian cells. These complexes are inhibited by cyclin/cdk inhibitor proteins such as p21^waf1^. The balance between the activation and inhibition of cyclin-cdk activities determines the cell cycle progression. Since cancer cells are characterized by abnormalities in cell cycle regulation, our findings indicate that p21^waf1^ may have a very similar function in both cancer cells and normal cells. It also underlies the possibility that in addition to the beneficial effects of butyrate and other HDAC inhibitors, which induce growth arrest in colon cancer, it may also cause some side-effects on normal cells.

**Figure 5:**
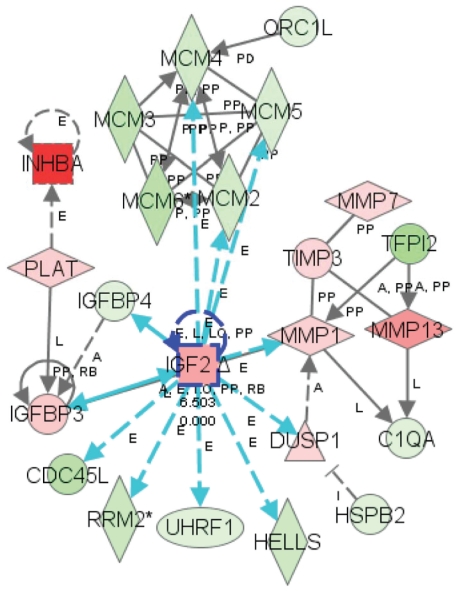
An integrated network reflecting the interaction of IGF2, extracellular matrix system and DNA replication Apparatus. Data set was analyzed by the Ingenuity Pathways Analysis software (Ingenuity^®^ Systems, www.ingenuity.com). The note color indicates the expression level of the genes (Red notes are up-regulated and green notes are down regulated). Notes and edges are displayed with various shapes and labels that present the functional class of genes and the nature of the relationship between the notes, respectively.

To determine the biologically relevant networks and pathways of the differentially expressed genes, pathway analysis was done using the Ingenuity Pathways Knowledge Base (Ingenuity^®^ Systems, www.ingenuity.com) (Li et al. 2006). One network identified contains up-regulated IGF2, IGFBP3, INHBA, MMP1, MMP13, etc., as well as down-regulated genes such as IGFBP4, MCM2, MCM3, MCM4, MCM5, MCM6, and ORC1L. In a graphical representation of the molecular relationships between genes/gene products (Network Pathway) (Fig. 5), genes or gene products are represented as nodes, and the biological relationship between two nodes is represented as an edge (line). All edges are supported by at least 1 reference from the literature, from a textbook, or from canonical information stored in the Ingenuity Pathways Knowledge Base. Our network analysis identified a complex network of genes involving IGF2, matrix metallo-proteases (MMP1 and MMP13), TIMP3, IGFBPs as well as genes for DNA replication that are regulated by butyrate in MDBK cells.

In conclusion, the present findings provide an example of epigenetic regulation of genome at work and a basis for understanding the full range of the biological roles and the molecular mechanisms that butyrate may play in human and animal cell growth, proliferation, and energy metabolisms. The results illustrated the potentialities of nutritional manipulation of gene activity for the purpose of enhancing animal health and production. This study has also generated comprehensive information on the experimental system that can be used in many functional genomic studies in bovine and provided a basis for understanding the full range of the biological roles and the molecular mechanisms that butyrate may play in animal cell growth, proliferation, and energy metabolism. More importantly, the present results suggest that Orc1, not Orc2 or Orc3, along with MCM proteins play a critical role in regulating the initiation of DNA replication and cell cycle progression in MDBK cells and are targets of butyrate regulation. All together, these findings highlight key molecular mechanisms by which butyrate induces G1 cell cycle arrest in normal bovine cells. The results illustrate the potential to exploit nutritional manipulation of gene activity to enhance animal production efficiency in a drug residue-free format.

## Figures and Tables

**Figure 1A:**
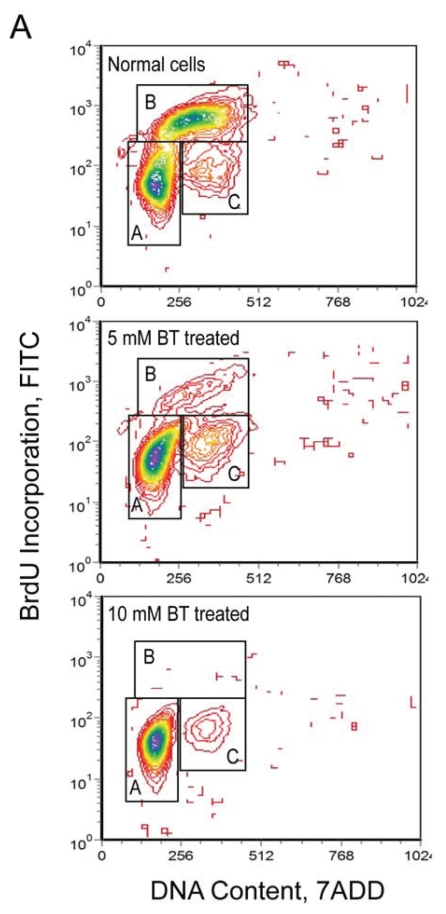
Cell population profiles determined by flow cytometry. For flow cytometric analysis, cells were first pulse labeled with BrdU for 30 min. Collected cells were first stained with diluted fluorescent (Fluorescent isothiocyanate, FITC) anti-BrdU antibody and then stained with DNA marker (7-ADD). The fluorescent signal generated by FITC was acquired in a logarithmic mode, and fluorescent signal from the DNA-content marker 7-ADD was normally acquired in the linear signal amplification mode. Cells were separated into three clusters by double staining analysis. **A**) G1/G0 cells, with 2C DNA content and without any DNA synthesis activity; **B**) S phase cells, with DNA content between 2C and 4C (two and four copies of DNA content respectively), and high BrdU incorporation (DNA synthesis) activity **C**) G2/M cells, with 4C DNA content and also without DNA synthesis activity.

**Figure 1B:**
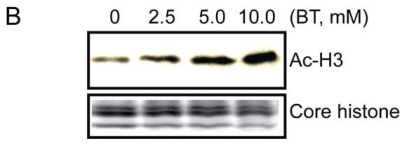
Butyrate induced hyperacetylation of histone 3 (H3). This figure is representative of three experiments. Protein from different samples was separated by SDS PAGE on two identical 4 to 20% polyacrylamide gradient gels. One gel was stained with SimpleBlue (Invitrogen) and one was transferred to a membrane for Western blotting with monoclonal anti-acetyl H3 antibodies.

**Table 1:** Primers Used in Real-Time RT-PCR.

Accession	Gene Symbols	Forward	Reverse
NM_174016	CDC2	TTCATGGGGATTCAGAAATTG	TTTCGAGAGCAGATCCAAG
NM_001046234	MCM6	TCAAGAAGAAGATGCCCAGATG	AGCCCAGCCTCAAGGAAG
TC293949	ORC1L	AGGAAGCAACATTTCAACAG	AGCATACAGCACATCATCC
NM_001038139	ORC3L	CCTTCGCCAACACCTGAATG	CCTCCGACCAGTCCACAAG
NM_001098958	CDKN1A	GCCCTTTCCCCTTAGTATCTTC	GTCGCTGCTTGAGGTAGAAC
NM_173979	ACTB	CCACCCCGCTTCTCTCTAAG	AATTTACACAAAAGCGATCACCTC

**Table 2:** Butyrate Down-regulated genes which products are directly related to initiation of DNA replication*.

Hugo gene symbol	Description	Mean fold change (n = 3)	False discovery rate, q-value	Location	Family
CDC2/CDK1	Cell division cycle 2, G1 to S and G2 to M	−8.217	0	Nucleus	kinase
MCM2	MCM2 minichromosome maintenance deficient 2, mitotin (S. cerevisiae)	−7.183	0	Nucleus	enzyme
MCM3	MCM3 minichromosome maintenance deficient 3 (S. cerevisiae)	−10.3	0	Nucleus	enzyme
MCM4	MCM4 minichromosome maintenance deficient 4 (S. cerevisiae)	−7.226	0	Nucleus	enzyme
MCM5	MCM5 minichromosome maintenance deficient 5, cell division cycle 46 (S. cerevisiae)	−6.002	0	Nucleus	enzyme
MCM6	MCM6 minichromosome maintenance deficient 6 (MIS5 homolog, S. pombe) (S. cerevisiae)	−11.618	0	Nucleus	enzyme
ORC1L	Origin recognition complex, subunit 1-like (yeast)	−4.368	0	Nucleus	other

* Data was extracted from microarray experiment, (Li and Li, 2006; Li et al. 2006).

**Table 3:** Butyrate-induced gene expression change in MDBK cells, validated by RT-PCR.

Genes	Control	Butyrate
CDC2	1.10 ± 0.54	0.15 ± 0.03
CDKN1A (p21)	1.07 ± 0.48	3.02 ± 0.68
MCM6	1.03 ± 0.30	0.46 ± 0.26
ORC1L	1.04 ± 0.38	0.19 ± 0.06
ORC3L	1.04 ± 0.36	1.24 ± 0.50
